# The Regional and Comparative Materia Medica of Drs. Malcolm and Moss

**Published:** 1895-09

**Authors:** 


THE REGIONAL AND COMPARATIVE MATERIA MEDICA of Drs. Malcom and Moss has been before the profession for several months. The editor of THE HOMŒOPATHIC PHYSICIAN has made continual use of it in his own practice, and cordially recommends it to the profession.

